# Three-dimensional evaluation of postoperative swelling in treatment of zygomatic bone fractures using two different cooling therapy methods: a randomized, observer-blind, prospective study

**DOI:** 10.1186/1745-6215-14-238

**Published:** 2013-07-29

**Authors:** Ali Modabber, Madiha Rana, Alireza Ghassemi, Marcus Gerressen, Nils-Claudius Gellrich, Frank Hölzle, Majeed Rana

**Affiliations:** 1 Department of Oral, Maxillofacial and Plastic Facial Surgery, University Hospital of the RWTH Aachen, Pauwelsstraße 30, Aachen 52074, Germany; 2 Department of Oral and Maxillofacial Surgery, Hannover Medical School, Carl-Neuberg-Strasse 1, Hannover 30625, Germany

**Keywords:** Zygomatic bone fracture, Three-dimensional optical scanner, Hilotherm, Conventional cooling

## Abstract

**Background:**

Surgical treatment and complications in patients with zygomatic bone fractures can lead to a significant degree of tissue trauma resulting in common postoperative symptoms and types of pain, facial swelling and functional impairment. Beneficial effects of local cold treatment on postoperative swelling, edema, pain, inflammation, and hemorrhage, as well as the reduction of metabolism, bleeding and hematomas, have been described.

The aim of this study was to compare postoperative cooling therapy applied through the use of cooling compresses with the water-circulating cooling face mask manufactured by Hilotherm in terms of beneficial impact on postoperative facial swelling, pain, eye motility, diplopia, neurological complaints and patient satisfaction.

**Methods:**

Forty-two patients were selected for treatment of unilateral zygomatic bone fractures and were divided randomly to one of two treatments: either a Hilotherm cooling face mask or conventional cooling compresses. Cooling was initiated as soon as possible after surgery until postoperative day 3 and was applied continuously for 12 hours daily. Facial swelling was quantified through a three-dimensional optical scanning technique. Furthermore, pain, neurological complaints, eye motility, diplopia and patient satisfaction were observed for each patient.

**Results:**

Patients receiving a cooling therapy by Hilotherm demonstrated significantly less facial swelling, less pain, reduced limitation of eye motility and diplopia, fewer neurological complaints and were more satisfied compared to patients receiving conventional cooling therapy.

**Conclusions:**

Hilotherapy is more efficient in managing postoperative swelling and pain after treatment of unilateral zygomatic bone fractures than conventional cooling.

**Trial registration:**

German Clinical Trials Register ID: DRKS00004846

## Background

The face represents the most prominent position in the human body and is often involved in trauma injuries. The zygomatic bone is particularly prone to facial injuries due to its prominence [1] and is the second most common mid-facial bone affected. The fracture of the zygomatic bone can pose considerable functional complications such as restricted mouth opening. Disruption of the zygomatic position can also carry psychological, aesthetic and functional significance, causing impairment of ocular and mandibular functions. Therefore, a prompt diagnosis of fracture and soft tissue injuries is important for both cosmetic and functional reasons [2].

In most cases the treatment of unilateral zygomatic bone fractures leads to a significant degree of tissue trauma that again causes an inflammatory reaction [3]. As a result, patients display common postoperative symptoms and types of pain, facial swelling and functional impairment [4]. Pain is typically brief and peaks in intensity in the early postoperative period. In contrast, facial swelling reaches the characteristic maximum 48 to 72 hours after surgery [5]. These symptoms can affect the patient’s quality of life and well-being. To increase patient satisfaction after treatment of uni- and bilateral zygomatic bone fractures, it is a necessary goal to minimize side effects as much as possible [6]. One way do so is to prescribe medication such as corticosteroids [7], non-steroidal anti-inflammatory drugs [8], a combination of corticosteroids and non-steroidal anti-inflammatory drugs [9] or enzyme preparations such as serrapeptase [10]. Furthermore, there are also nonmedication methods to treat the above side effects. These can include manual lymph drainage [11], soft laser [12,13] and cryotherapy [14]. Historically, the therapeutic use of local or systemic cryotherapy was first described by Hippocrates [15]. Beneficial effects of cold treatment on postoperative swelling have been described previously [16-20] as well as the positive impact on edema, pain and inflammation [21-23]. The activity of inflammatory enzymes rises with increasing temperatures [21]. On reviewing the literature, there is a lack of scientific evidence and trials in oral and maxillofacial surgery which show positive as well as no effect of cold therapy [24]. Cooling therapy varies from the conventional, such as ice packs, gel packs or cold compresses, to mechanically supported continuous cooling with face masks. Both positive and negative side effects have been previously discussed [16,20]. The aim of this study was to examine the effect of hilotherapy in comparison with a conventional cooling method using cold compresses on swelling, pain, eye motility, diplopia, neurological complaints and overall patient satisfaction following treatment of unilateral zygomatic bone fractures.

## Methods

The study was approved by the local ethics committee at the University Aachen, Germany (EK 142/2008). Before the beginning of the study, written informed consent was obtained from each patient.

### Patients

**Figure 1:**
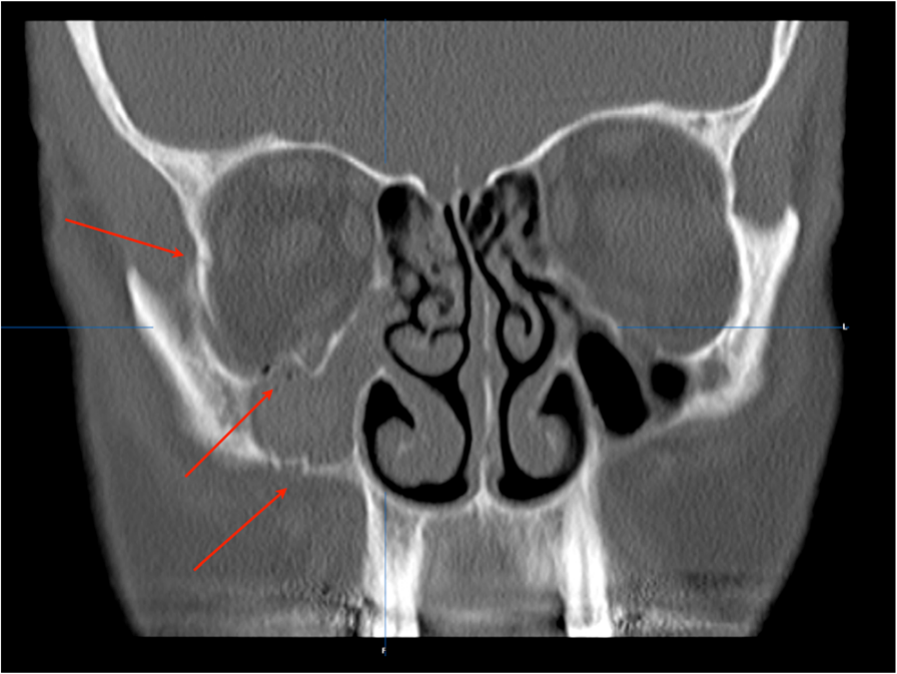
**The coronal view of a 24-year-old patient shows an isolated zygomatical fracture on the right side.** Red arrows demonstrate the fracture lines.

Forty-two healthy patients were scheduled for treatment of unilateral zygomatic bone fractures (Figure 1). Only patients who required open reduction and internal fixation using a 3 point fixation technique were divided randomly into two treatment groups. One group of 21 patients were treated with conventional cooling and the other group of 21 patients received continuous cooling using hilotherapy after repositioning of unilateral zygomatic bone fractures. The observer was not aware of the kind of therapy that was applied at the time of the patient examinations and during analysis of the data. The patients were not blinded and were informed that the study was designed to compare the effect of the Hilotherm cooling face mask and conventional cooling compresses on swelling, pain, eye motility, diplopia, neurological complaints and patient satisfaction.

### Fixation methods

**Figure 2:**
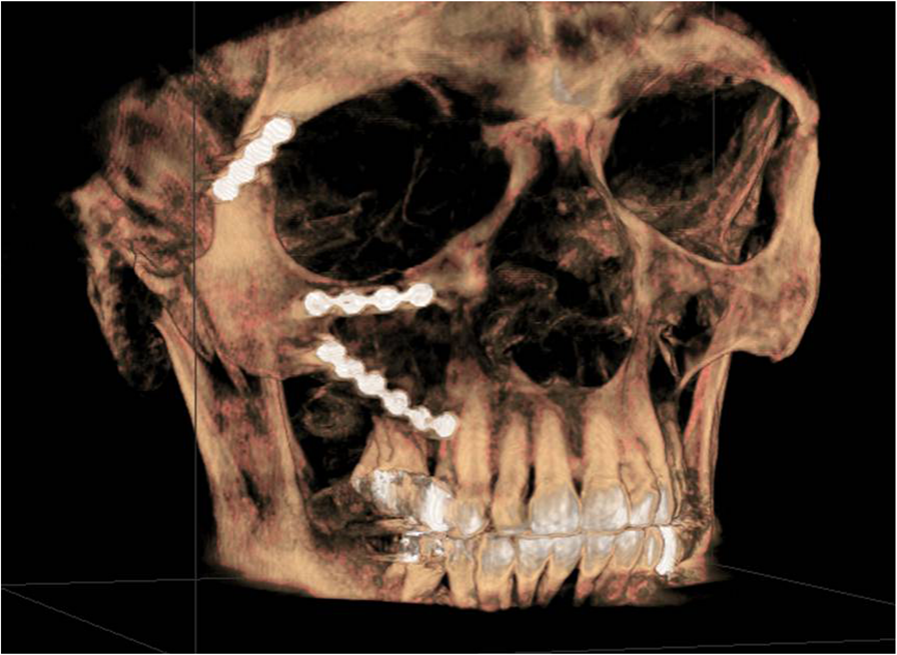
Three-dimensional reconstruction of postoperative cone beam computed tomography after osteosynthesis of a right-side zygomatical fracture, along the frontozygomatic suture, infraorbital margin and zygomatico maxillary buttress.

The fracture sites were exposed using different standard incisions. Frontozygomatic suture was approached using an eyebrow incision, zygomatico maxillary buttress was exposed using an intraoral buccal sulcus incision and additional exposure of the infraorbital rim was accomplished using an infraorbital approach. In all cases, plating was attempted along frontozygomatic suture, infraorbital margin and zygomatico maxillary buttress (Figure 2). The osteosynthesis was performed with 2.0 mm or 1.5 mm plates (Stryker, Duisburg, Germany) per fracture line.

### Cooling methods

**Figure 3:**
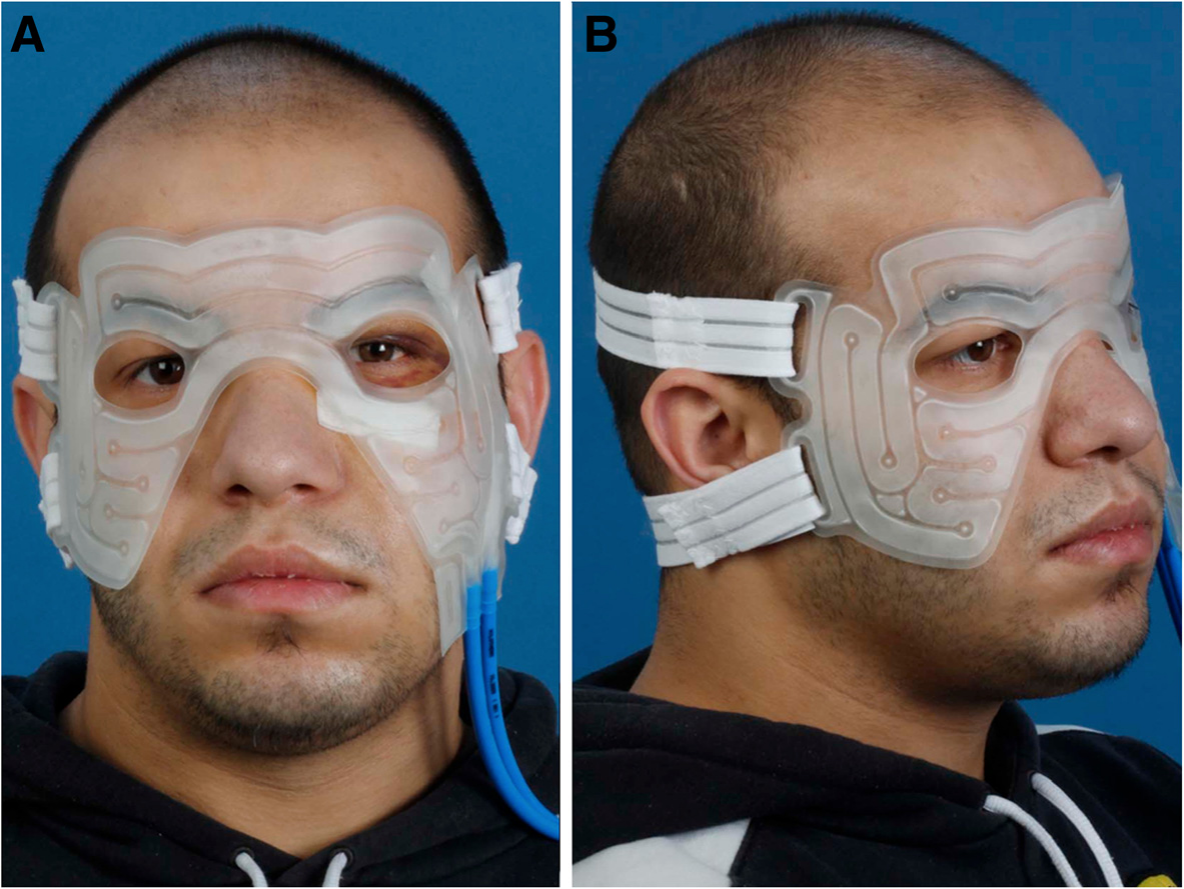
Front view (A) and lateral view (B) of a patient wearing the Hilotherm mask.

Hilotherapy refers to the water-circulating external cooling device Hilotherm Clinic (Hilotherm GmbH, Argenbühl-Eisenharz, Germany) that consists of a preshaped thermoplastic polyurethane mask and the Hilotherm cooling device control unit (Figure 3A,B). The temperature setting is adjustable from +10°C to +30°C and was set to 15°C immediately after surgery. Conventional cooling was performed through cool compresses. Both cooling methods were initiated as soon as possible after surgery until postoperative day 3 continuously for 12 hours daily.

### Study protocol and inclusion criteria

Only patients with a unilateral zygomatic bone fracture were included in this study. Potential participants were excluded from the study because of missing operability, the possibility of missing the follow-up examination, pregnancy, nursing, drug addiction, recent operations, diseases of the heart, metabolism and central nervous system, infectious disease, and diseases affecting the circulation, systemic, malignant and immune systems, as well as blood coagulation disorders and allergic reactions to pharmaceuticals and antibiotics. The clinical inclusion and exclusion criteria are shown in Table 1. All patients were examined and scanned on fixed dates using standardized methods and techniques. Thus, each patient received the same postoperative analgesic drug therapy which included 1000 mg paracetamol intravenously twice daily for 3 days, 600 mg ibuprofen orally (day 1, ibuprofen 600 mg three times per day; day 2, ibuprofen 600 mg twice daily; day 3, ibuprofen 600 mg once daily; day 4, ibuprofen 600 mg once daily). Antibiotic prophylaxis consisted of 600 mg clindamycin intravenously three times daily for 3 days. A single perioperative dose of 250 mg steroids was administered to each patient intravenously. During a first visit, the physician collected information about past illnesses and diseases and conducted a standard blood test. The operation took place using general anesthesia and oral intubation.

**Table 1:** Study inclusion and exclusion criteria

**Inclusion criteria**	**Exclusion criteria**
Unilateral zygomatic fracture	Complex midfacial fracture
Combination of infraorbital approach, eyebrow and buccal sulcus incision	Panfacial fracture
Osteosynthesis using 2.0 mm and 1.5 mm plates (Stryker)	Polytrauma
Plating along frontozygomatic suture, infraorbital margin and zygomatico maxillary buttress	Infected fractures
Age between 18 and 79 years	Pathological fractures
Written informed consent	Missing operability
	Potential to miss the follow-up examination
	Pregnancy
	Heart, pulmonary, liver and kidney disease, chronic pain syndrome
	Drug addiction
	Recent operations,
	Diseases affecting metabolism, central nervous system, infectious, circulation, systemic, malignant and immune system
	Blood coagulation disorders
	Allergic reactions to pharmaceuticals and antibiotics
	Dermatological diseases of the face
	Raynaud´s phenomenon

During the study the following parameters were assessed: pain, swelling, eye motility, diplopia, neurological complaints and patient satisfaction. To minimize bias through patient contact, the patients were examined and hospitalized in separate rooms.

### Measurement of facial swelling

**Figure 4:**
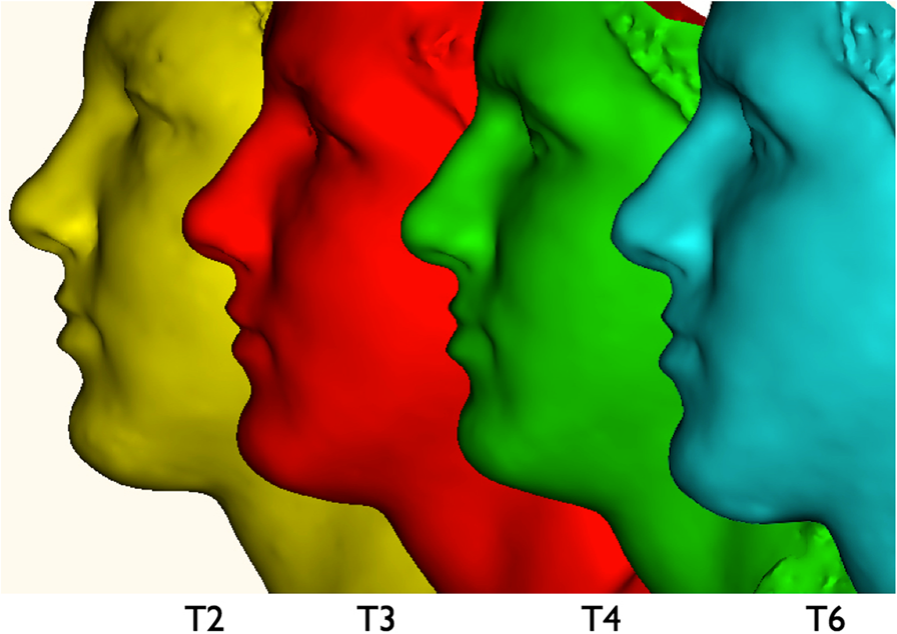
**The final three-dimensional output of the Slim3D software is a triangulated polygon mesh, visualized as a synthetically shaded representation.** Three-dimensional optical scans were recorded during six time points: T1 (day 1 after surgery, mask not shown), T2 (day 2 postoperatively, yellow mask), T3 (day 3 postoperatively, red mask), T4 (day 7 postoperatively, green mask), T5 (day 28 postoperatively, mask not shown) and T6 (day 90 postoperatively, blue mask). The reference three-dimensional model of each patient was T6. An individual mask of the midface of each patient was created and aligned to all captures and the difference in volume was thereby calculated.

This study used the three-dimensional optical scanner, FaceScan3D (3D Shape GmbH, Erlangen, Germany), to measure facial swelling in volume (ml) as described previously [18-20]. The three-dimensional optical scanner consists of an optical range sensor, two digital cameras, a mirror construction and a commercial personal computer. The sensor is based on a phase-measuring triangulation method [25]. There is no need for special safety precautions for the patient, since the advantage of this optical sensor is its contactless data acquisition accompanied by its high accuracy in the z-direction with 200 μm and a short measurement time of 430 ms. The mirror construction permits the capture of over 180° of the patient’s face. The computer program Slim 3D (3D Shape) automatically triangulates, merges and postprocesses the data [26]. The final output is a triangulated polygon mesh that is visualized as a synthetically-shaded or wire-mesh representation [27]. For the volume calculation all patients were photographed with a standard technique for frontal views of the face. Adjustment occurred on the Frankfurt horizontal line, parallel to the floor. Patients sat on a self-adjustable stool and were asked to look into a mirror with standard horizontal and vertical lines simulating a red cross marked on it. The horizontal line was adjusted to subnasale and the vertical line was aligned to the midline of the face. Patients were instructed to swallow hard and to keep their jaws in a relaxed position for the scan. Three-dimensional optical scans were recorded at six points in time: on day 1 after surgery (T1), on day 2 (T2), day 3 (T3), day 7 (T4), day 28 (T5) and day 90 (T6) postoperatively . For each patient we chose time point T6 as a reference, because at this time point swelling of soft tissue could be excluded which otherwise could influence the measurements. Annotations of T1 to T6 were prepared by an error minimization algorithm which applied modified Iterative Closest Point using simulated annealing by the Levenberg-Marquardt algorithm [28,29]. To minimize disturbance of soft tissue during the registration process only facial areas that were not influenced by the swellings were used for surface matching: the forehead, ears and root of the nose. The geometrical models were aligned with the forehead and the ears. After the aligned shell deviation panels were created for cutoff to create an individual mask of the face (Figure 4).

### Pain analysis

Postoperative pain analysis was conducted with the help of a 10-point visual analogue scale based on measurements before surgery (T0), on day 1 (T1), day 2 (T2) and day 7 (T3) postoperatively, where the patients had to rate their pain on a score from 0 to 10, with 0 describing a situation without pain and 10 denoting a maximum intensity of pain.

### Neurological analysis

The neurological analysis was utilized in order to enable the evaluation of nerve dysfunctions. The results were recorded on a score that ranges between 0 and 9, with 9 being the worst neurological score. The skin of the upper lip was checked using a cotton test for touch sensation (regular = 0; hypesthesia = 1; anesthesia = 2), a pinprick test using a needle for sharp pain (regular = 0; hypalgesia = 1; analgesia = 2), and a blunt instrument for testing sharp-blunt-discrimination (regular = 0; partly = 1; none = 2). Additionally, a two-point discrimination test (0 to 0.9 cm = 0; 1 to 2.5 cm = 1; 2.6 to 4 cm = 2; >4 cm = 3) was executed on the lip. The neurological score was assessed at five points in time: before surgery (T0), on day 1 (T1), day 7 (T2), day 28 (T3), and day 90 (T4) postoperatively.

### Eye motility and diplopia

For the analysis of eye motility and diplopia the patient was required to fix on a light source at a distance of 30 cm. While the head was fixed, the light source was guided in different directions of view. The relative displacement of the reflected images to each other and the movement of the eye were analyzed. Meanwhile, the patient was asked about diplopia. The data were collected at four points in time: before surgery (T0), on day 1 (T1), day 7 (T2) and day 28 (T3) postoperatively.

### Patient satisfaction

Each patient was asked to complete a questionnaire on the postoperative day 10, subjectively rating their comfort and satisfaction with the applied postoperative cooling therapy. The grading scale ranged from 1 to 4, where 1 denoted “very satisfied” and 4 “not satisfied”.

### Statistical analysis

To check for statistical significance of quantitative variables, the Student *t*-test for unrelated samples was used. All data are expressed as mean values ± standard deviation, with a *P*-value ≤0.05 taken as significant. For analyzing gender, eye motility and diplopia, a *χ*^2^-test was utilized, and a *P*-value ≤ 0.05 was taken as a level of significance. The statistical analysis was conducted using SPSS for Windows version 14.0 (SPSS Inc., Chicago, IL, USA).

## Results

### Baseline characteristics

Forty-two patients were randomly enrolled in the study. After reposition and osteosynthesis of unilateral zygomatic bone fractures, 21 patients were assigned to conventional cooling therapy and 21 patients were treated with hilotherapy. The clinical and demographic characteristics of patients in both groups are shown in Table 2. Both groups showed no statistical significances regarding gender, age, body mass index, surgery duration, hospitalization duration, preoperative pain and neurological score as well as preoperative limited eye motility and diplopia.

**Table 2:** Baseline characteristics of patients

	**Hilotherm**	**Conventional**	***P-*value**
Female gender (n/total (%))	4/21 (19)	3/21 (14)	0.68
Age (years)	36.5 ±16.1	35.6 ± 21.9	0.89
Body mass index (kg/m^2^)	23.8 ± 3.6	24.4 ± 3.8	0.56
Surgery duration (minutes)	70.2 ± 33.4	73.9 ± 38.7	0.74
Hospitalization duration (days)	4.6 ± 1.9	4.4 ± 1.1	0.69
Preoperative pain score (visual analogue scale)	3.1 ± 0.7	3.2 ± 0.8	0.55
Preoperative neurological score	3.4 ± 1.7	3.5 ± 1.7	0.86
Preoperative limited eye motility (n/total (%))	12/21 (57)	13/21 (62)	0.75
Preoperative diplopia (n/total (%))	10/21 (48)	10/21 (48)	1.00

All values are ± standard deviation unless indicated otherwise.

### Postoperative swelling

**Figure 5:**
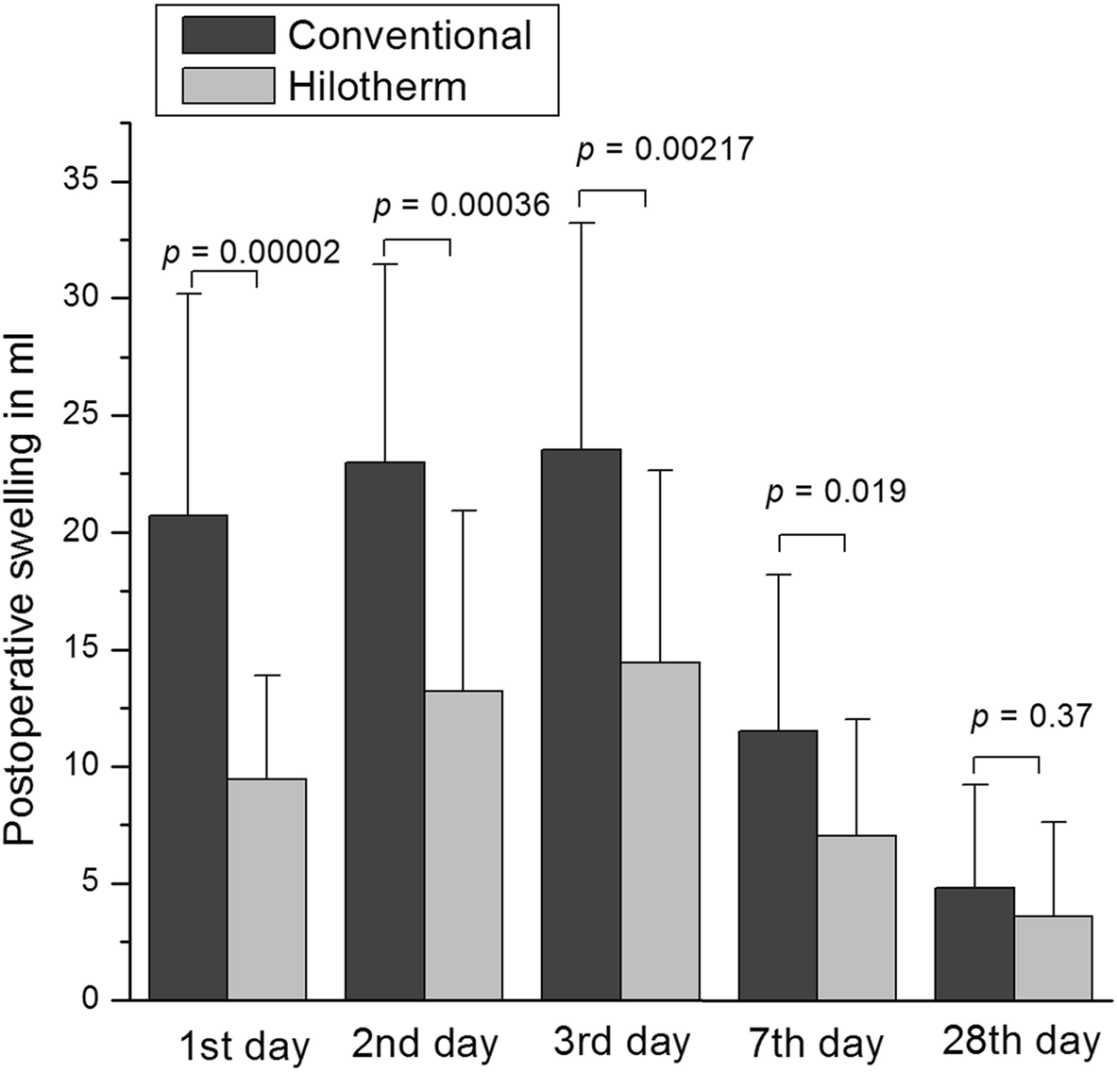
**The amount of swelling (ml) in both groups at different time points is shown.** On postoperative days 1, 2 and 3, a significant downregulation of swelling could be achieved by cooling with Hilotherm compared to conventional cooling. This trend was maintained on postoperative day 7. After 28 days no differences with respect to swelling could be seen between groups.

Swelling was measured in terms of volume (ml) as described in the methodology section. On the day 1 following surgery a statistically significant reduction in swelling could be seen by applying the Hilotherm cooling device compared to conventional cooling therapy (Hilotherm 9.45 ± 4.42 ml versus conventional 20.69 ± 9.05 ml, *P* = 0.00002) (Figure 5). Maintaining this tendency on day 2 following surgery, a statistically significant reduction in swelling could be seen (Hilotherm 13.20 ± 7.71 ml versus conventional 22.97 ± 8.50 ml, *P* = 0.00036). On day 3 (Hilotherm 14.44 ± 8.21 ml versus conventional 23.52 ± 9.69 ml, *P* = 0.00217) and on day 7 (Hilotherm 7.06 ± 4.97 ml versus conventional 11.51 ± 6.70 ml, *P* = 0.01907) the measured swelling was also significant. On the postoperative day 28, the measured swelling was almost equal in both groups (Hilotherm 3.62 ± 4.02 ml versus conventional 4.80 ± 4.43 ml, *P* = 0.36980). Maximal swelling was noticed on postoperative day 3 (Figure 5).

### Postoperative pain score

**Figure 6:**
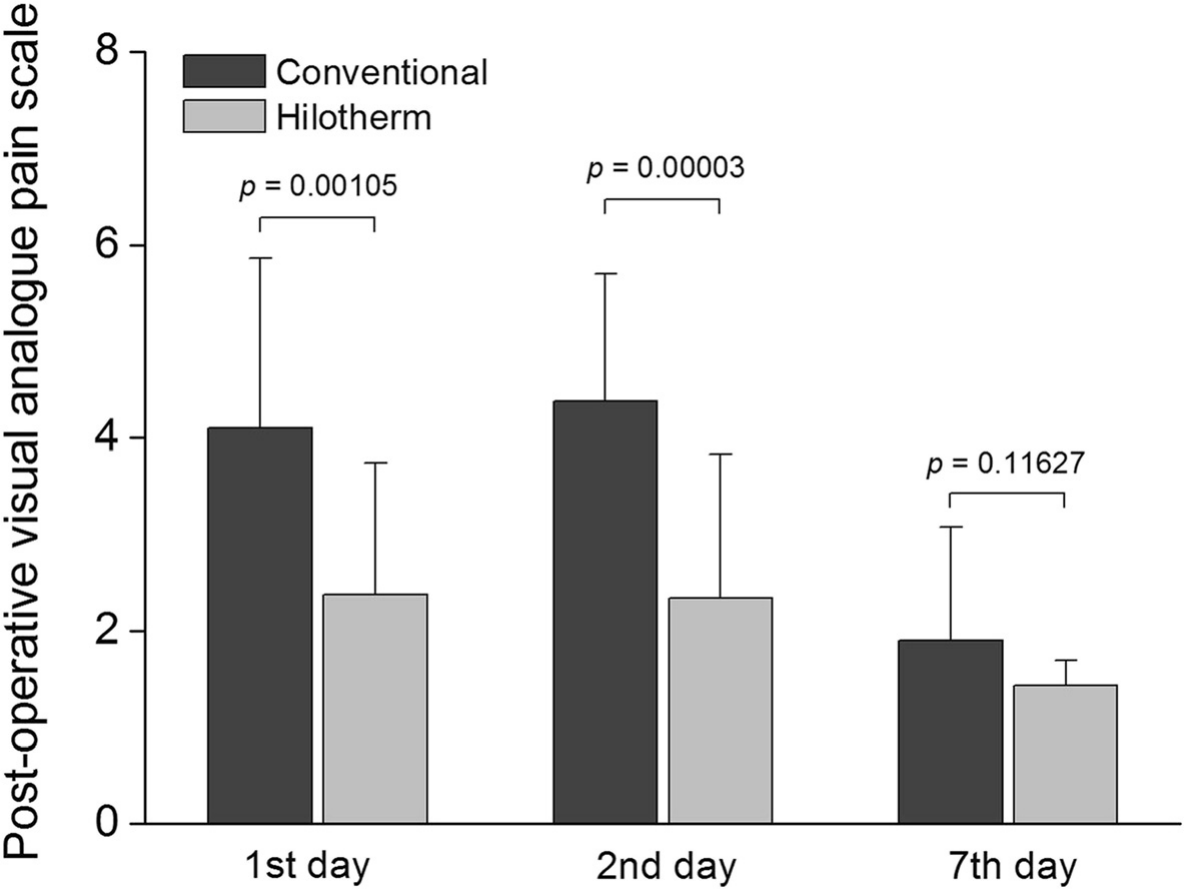
**Pain was calculated in terms of a visual analogue scale from subjective analysis ranging from 0 to 10.** A significant increase in pain was reported in the conventional group compared to the Hilotherm group during postoperative days 1 and 2. The pain intensity was no different between groups on postoperative day 7.

Pain was quantified in terms of a 10-point visual analogue scale ranging from 0 to 10, based on subjective analysis. On postoperative days 1 and 2, a significantly reduced pain score was obtained by hilotherapy compared to conventional cooling (day 1, Hilotherm 2.38 ±1.36 versus conventional 4.10 ± 1.76, *P* = 0.00105; day 2, Hilotherm 2.34 ± 1.49 versus conventional 4.38 ± 1.32, *P* = 0.00003). No statistically significant difference could be seen on postoperative day 7 (Hilotherm 1.43 ± 0.68 versus conventional 1.90 ± 1.18, *P* = 0.11627) (Figure 6).

### Postoperative neurological score

**Figure 7:**
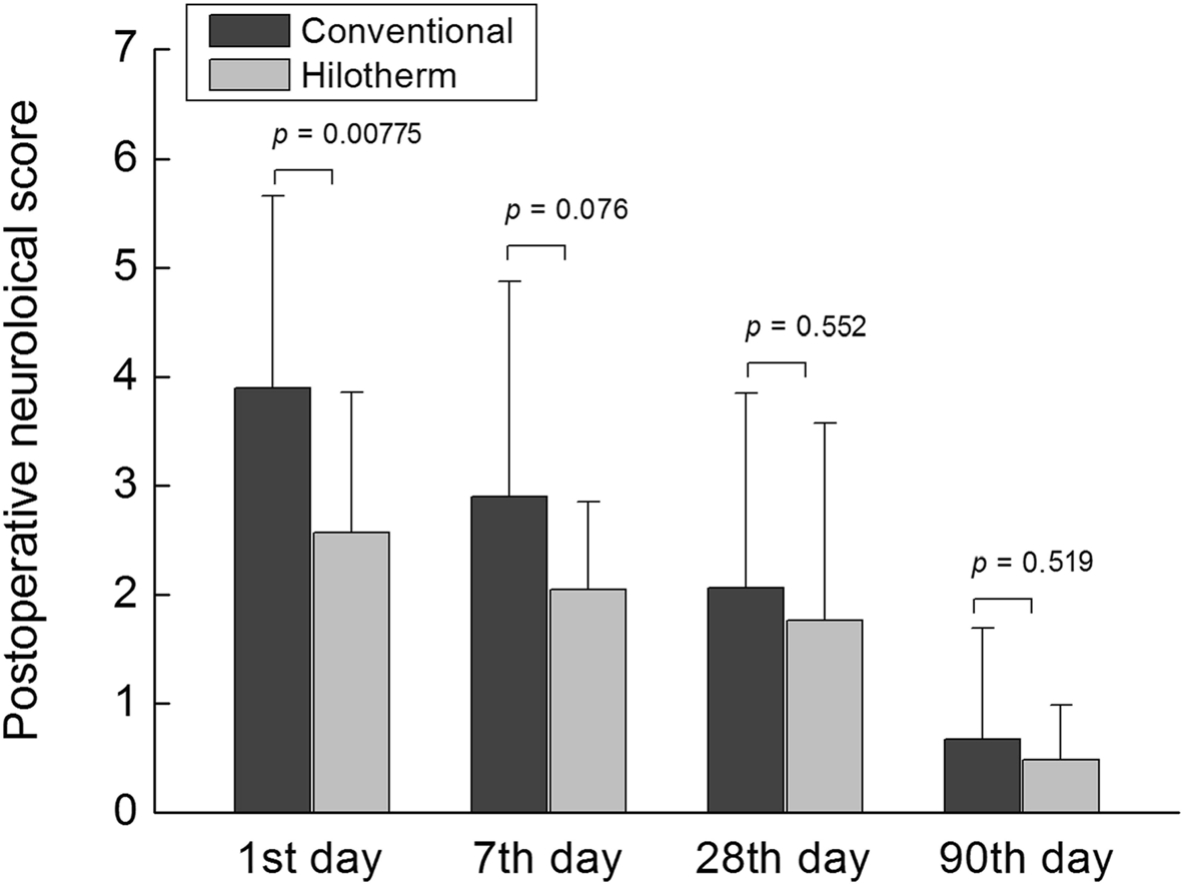
Reduction was seen in the Hilotherm group in the neurological score at postoperative day 1, but no differences were detected after 7, 28 and 90 days between groups.

Hilotherapy obtained a significantly reduced neurological score at day 1 compared to conventional cooling (Hilotherm 2.57 ±1.29 versus conventional 3.90 ± 1.76, *P* = 0.00775). There were no statistically significant differences between groups concerning the neurological score at postoperative days 7, 28 or 90 (day 7, Hilotherm 2.05 ± 0.80 versus conventional 2.90 ± 1.97, *P* = 0.07642; day 28, Hilotherm 1.76 ± 1.81 versus conventional 2.06 ± 1.79, *P* = 0.55187; day 90, Hilotherm 0.48 ± 0.87 versus conventional 0.67 ± 1.02, *P* = 0.51947) (Figure 7).

### Eye motility and diplopia

Using a *χ*^2^-test, no statistically significant differences were found preoperatively between groups with respect to eye motility and diplopia (Table 2). On postoperative day 1, a significant reduction in eye motility limitation (Hilotherm, 17 patients without and 4 patients with limited eye motility versus conventional, 11 patients without and 10 patients with limited eye motility, *P* = 0.050) and diplopia (Hilotherm, 18 patients without and 3 patients with diplopia versus conventional, 11 patients without and 10 patients with diplopia, *P* = 0.019) was obtained through hilotherapy compared to conventional cooling. There were no statistically significant differences found between groups concerning the limitation of eye motility and diplopia 7 and 28 days after surgery (day 7, Hilotherm, 18 patients without and 3 patients with limited eye motility versus conventional, 15 patients without and 6 patients with limited eye motility, *P* = 0.259; Hilotherm, 19 patients without and 2 patients with diplopia versus conventional, 16 patients without and 5 patients with diplopia, *P* = 0.214; day 28, 19 patients without and 2 patients with limited eye motility in both groups, *P* = 1.000; 20 patients without and 1 patient with diplopia in both groups, *P* = 1.000).

### Patient satisfaction

**Figure 8:**
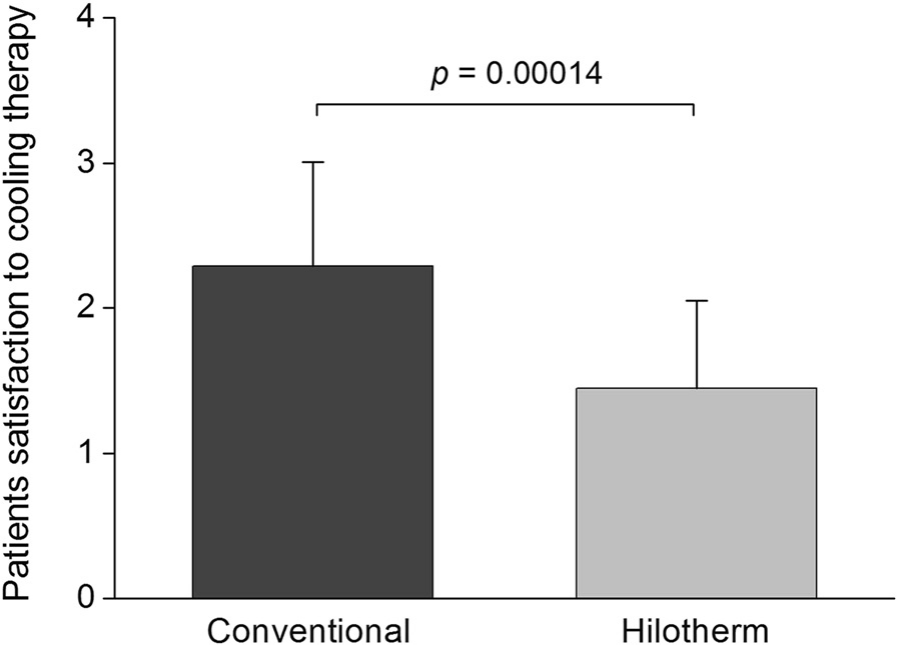
The overall satisfaction was significantly lower in patients receiving conventional therapy compared to patients receiving cooling therapy by Hilotherm.

Regarding patient satisfaction, which was assessed at day 10 after surgery, a statistically significant difference between hilotherapy and conventional cool packs could be detected. Patients treated with hilotherapy had a significantly greater satisfaction (Hilotherm 1.43 ± 0.60 versus conventional 2.29 ± 0.72, *P* = 0.00014) (Figure 8).

## Discussion

This study demonstrates that continuous cooling with the hilotherapy device reduces postoperative swelling and pain in the treatment of unilateral zygomatic fractures compared to conventional cooling with cold packs. Furthermore, satisfaction of patients treated with hilotherapy was greater compared to patients who received conventional cooling. However, eye motility limitation, diplopia and neurological score revealed significant differences only at postoperative day 1. Wound healing was uneventful. Malfunctioning of the Hilotherm cooling device did not occur.

The healing process and possible complaints regarding the treatment of facial trauma can be influenced by patient-related factors such as age and gender, compliance and health status as well as patient independent factors such as surgeon experience, duration of surgery time, extent of trauma and fragment dislocation as well as use of antibiotics [3,18,19,30]. Since in this study the use of antibiotics and the duration of surgery time were not significantly different among both groups, and since health-compromised patients were excluded from the study, these factors are considered not to have influenced the observed results.

Although the effects of different cooling methods have been investigated for a number of maxillofacial and plastic surgery treatment procedures, there is so far no study comparing conventional cooling versus hilotherapy following treatment of zygomatic bone fractures [18,19,31-33].

Consistent with our results, Belli and colleagues [31] reported the safe use of hilotherapy as well as a postoperative decrease in pain and swelling intensity and duration after Le-Fort-I osteotomy and bilateral sagittal osteotomy of the lower jaw. While they investigated only 10 patients without a comparison to other cooling techniques, Jones and colleagues [32] recorded differences between hilotherapy and conventional groups in a greater cohort of 50 patients following face-lift surgery procedures. In contrast to our results, Jones and colleagues [32] described a statistically significant increase in patient-reported postoperative swelling in the Hilotherm group with no significant differences regarding ecchymosis, hematoma or pain between groups. However, subjectively the majority of patients found the cooling masks to be comforting. In order to overcome the lack of significance of subjective assessments versus objective evaluation methods, Moro and colleagues [33] measured the distance of multiple anatomic landmarks for swelling purposes. In so doing, 90 patients operated on for maxillomandibular malformations were divided into three groups and treated either with hilotherapy, conventional cooling or left untreated as a control group. As expected, no cryotherapy treatment led to the worst results whereas cooling with the hilotherapy method showed the least degree of swelling.

With the aim of improving measurement accuracy of different swelling stages, our study group used three-dimensional evaluation by the means of an optical face scanner [18-20]. Hence, three-dimensional volumes could be measured instead of two-dimensional lines.

Although cryotherapy is a relatively safe way to treat complications after oral or maxillofacial surgeries, cold therapy should only be employed with caution. Above all, very young or very old patients can react with intolerances to external cooling [34].

Topographical considerations make it difficult to quantify the facial volume of swelling. However, there are some limitations of this measurement technique which have to be discussed. The volume measurement with this technique is limited to localized facial swelling, since facial areas which have not been affected by the swelling are necessary for surface matching [18,19]. Some methods are described to predict soft tissue via cephalograms, which are able to create three-dimensional images. Ethically, the benefit of cephalograms might not justify the patient’s exposure to ionizing radiation [35].

In summary, use of the cooling device by Hilotherm reduces postoperative swelling and pain compared to conventional cooling. Biological effects of cooling therapy on vascular, neural, metabolic and muscular sites are known. Cryotherapy decelerates cell metabolism because, according to Van’t Hoff law, it slows down biochemical reactions. Regarding vascular effects, cold therapy constricts blood vessels. The intensity of vasoconstriction reaches the highest value at a temperature of 15°C. Furthermore, a decrease in body temperature slows down peripheral nerve conduction. For temperatures below 15°C, nerve conduction is completely disabled and the vasoconstriction turns into a vasodilatation. These biological effects influence postoperative symptoms. Meanwhile, the anti-edema effect is caused by the vasoconstriction and the pain reducing effect of the cold is related to a blocking of nerve endings. This blocking decelerates nerve conduction, and consequently the inflammation phenomena. Ice packs or similar conventional cooling methods use a temperature of around 0°C. Such a low temperature constrains lymph drainage and cell metabolism [36]. The effects of a treatment with overly low temperatures have already been mentioned. The inference is that a system is needed that maintains the desired temperature over a fixed period of time. To fulfill this requirement, this study worked with the cooling device Hilotherm Clinic (Hilotherm GmbH) [37]. Further studies are needed to investigate the benefits of this technique in other clinical research areas.

## Conclusions

Hilotherm is easy to use for both, patients and medical staff. Constant cooling with the possibility of adjusting temperature are important advantages. This is why hilotherapy is expected to play a greater role in oral and maxillofacial surgery as well as other clinical fields in the future.

### Ethical approval

Approval for the study was obtained from the relevant ethics committee at the University of Aachen, Germany (EK 142/2008). Before the beginning of the study, written informed consent was obtained from each patient. The study was registered with the Trial Registration Number: DRKS00004846.

## Competing interests

The authors declare that they have no competing interests.

## Authors’ contributions

AM and MR were responsible for the study concept and design. AM was responsible for data acquisition and writing the paper. AM and MADR carried out the statistical analysis. All authors were responsible for data analysis and interpretation. AM and MR drafted the manuscript. MR, FH, NCG, AG and MG were involved in revising the manuscript. All authors reviewed the manuscript. All authors read and approved the final manuscript.
